# Exploring the career maze: An investigation of career intentions of medical graduates in Pakistan: A qualitative study

**DOI:** 10.12669/pjms.35.5.465

**Published:** 2019

**Authors:** Zarrin S. Siddiqui

**Affiliations:** Dr. Zarrin S. Siddiqui, PhD. MD Education Unit, The University of Western Australia, Perth, Australia

**Keywords:** Career intentions, Medical education, Pakistan, Postgraduate trainees, Theory of planned behaviour

## Abstract

**Background & Objectives:**

Pakistan faces a number of challenges in medical education. While there is an increase in the number of medical schools across the country, there is a dearth of practicing doctors in rural areas as well as a shortage of specialists in various fields specially in surgical specialties. Similarly, the number of doctors migrating overseas is also increasing due to security concerns. This requires investigation of the factors that influence career intentions of medical graduates in Pakistan. As there is no validated instrument available within Pakistani context, this qualitative study was designed to examine medical graduates’ reasons for their career intentions in light of Ajzen’s Theory of Planned Behavior (TPB).

**Methods:**

Five focus group discussions were conducted in two cities of Pakistan i.e. Karachi and Hyderabad during November – December 2012. These were then transcribed and were coded into the three primary attributes of TPB i.e. behavioral, normative and control beliefs by comparing similarities and differences.

**Results:**

The results suggest that there is a variation in the factors that influence the career intentions of the medical students. In addition a number of new themes were identified which have not been reported earlier in studies elsewhere and are specific to this region. This needs further examination by stakeholders for intervention.

**Conclusion:**

The analysis of data from the focus groups confirms the theoretical framework and identifies a range of influencing factors, at different stages of education and practice. As the study was limited to a smaller number of graduates and all except one graduate wanted to continue practice, a larger sample may be required for the purpose of generalization of the findings reported in this study.

## INTRODUCTION

Pakistan faces a number of challenges in medical education. These include increased number of medical schools with majority of female students. In addition, overseas migration of doctors has worsened the situation with shortage of specialists as well as general practitioners especially in the rural areas.

It is commonly observed that a large proportion of female medical graduates do not practice after graduation let alone go for specialization.^1^,^2^ Therefore a need to closely examine the career intentions of medical graduates and its impact on workforce is required. The objective of this study was to identify the factors that influence the career intentions of medical graduates.

The theoretical framework used for this study is the Ajzen’s Theory of Planned Behavior which proposes that intention to perform a behavior is the central determinant of behavior.^3^

The Theory of Planned Behavior has three constructs that lead to the formation of the intention.^4^-^7^ These include;

- *Behavioural beliefs/attitudes:* These are beliefs about the likely consequences of behaviour and TPB suggests that this construct is directly related to one’s intention to perform a behaviour i.e. the more favorable one’s views are about a particular behaviour; the more chances are that one will have the intention to perform that behaviour.
- *Normative beliefs/subjective norms:* These are beliefs about normative expectations of others and consider the social pressures that a person faces in order to perform or not to perform behaviour.
- *Control beliefs:* These are beliefs about the presence of factors that may facilitate or impede the performance of that behaviour. This construct relies on the experiences of an individual that they encounter in attempting a particular behaviour.

## METHODS

Five focus group discussions were conducted by the author in Karachi and Hyderabad during November - December 2012 at their workplace, following ethics approval from the UWA Human Research Ethics Committee. The sessions were attended by house officers and postgraduate trainees. At the beginning of each session, participants were briefed about the research and the researcher’s background. Those who wished to take part in the discussions were asked to sign the consent. The questions used in the discussions were developed through discussion with other medical educators in Pakistan. These explored;

- The motivation to enter the field of medicine and what facilitated or constrained the motivation during the medical course.
- The career intention at the time of entry to medicine and whether it changed during the course of studies.
- The satisfaction with the choice the participants have made.
- The factors that need to be considered to increase the retention of medical graduates in workforce.

Once all the data was transcribed, the responses were de-identified and a code was assigned to each participant. Thematic analysis was used to explore the career intentions of the medical graduates by identifying the themes. The steps used in the analysis follow the six– step approach identified by Braun to generate themes from open coding.^8^

Initially data were coded into the three primary attributes of TPB by comparing similarities and differences. To assess the strength of each belief, the whole experience of medical course was divided into three stages i.e at the time of entry into medical course (Pre-exploration stage), during the medical course (Exploration stage) and after graduation (Stabilization stage). Finally, themes were created using all of the existing categories to identify factors that were felt to be important for the retention of medical graduates in the workforce in Pakistan. The credibility of analyses was enhanced through one observer present in the focus group discussion who later went through the emergent codes and classification into themes. Additionally two academics outside Pakistan were included in the analysis and generation of codes independently.

## RESULTS

In all, thirty-two graduates participated in group discussions. Each discussion lasted from 90 minutes to two hours. The profile of the participants is shown in Table-I. Results are presented in three stages with themes. Sample comments are presented for each theme with a two digit identification code and gender of the participant i.e. F for female and M for male participants.

**Table I:** Profile of the participants (n=32).

Characteristics	N=32
Mean age (Year & SD)	27 years +/- 4
***Gender***	
Male	10
Female	22
***Current status***	
House officer	17
Postgraduate trainee/RMO	15
***Place of Graduation***	
Public	30
Private	02

### Pre-exploration Stage

In the first stage, the participants’ motivation for entering the medical course was explored. The primary motive for the majority of the participants (n= 22) i.e. 69% was personal interest and/or family influence. When explored further, the reasons provided for their motivation to enter were mainly behavioural beliefs i.e. to serve the humanity, interest in science subjects or vice versa, no doctor available in their area or family, independence and for financial gains etc.

### Family Pressure

*He (my father) wanted that one of his children should become a doctor. It was not my wish. I am second among siblings. My older sister was not at all interested in education so I have to be a doctor*. ***RA(F)***

The participants identified a range of normative beliefs. These include enhancing the social status, a safe and respectable profession for girls and the presence of a role model in the family. Eight participants had no interest in medicine but enrolled into the medical school because of the family’s influence or because they thought, other subjects were harder.

None of the comments were categorized into control beliefs during this stage.

### Exploration Stage

In this section, the participants were asked about the effect on motivation during the course, and what facilitated or constrained their motivation. Time was the main reason that respondents identified as affecting their motivation.

The course itself is very lengthy and it does not finish in time. Year one took one and half year to complete and same with final year and so your friends who have opted for some other subjects actually graduate much earlier than you. It takes 7 – 8 years. Then there is a gap of seven months between results and starting the house job. **AA (M)**

Respect and social status continued to show up as recurring themes and have a positive effect on the motivation. When students enter the clinical rotations, they experience the respect shown to them by the patients and their attendants. At the same time, they also get special attention in the family.

…you see patient giving you all their blessings so you feel lucky that you belong to a noble profession where every moment is an act of kindness. Patients’ blessings and then family saying that their son is a doctor, all of these have increased my motivation. **UD(M)**

Four more themes emerged in this stage. These are Finances/Resources, Politics, Safety and the Learning environment.

### Finances/Resources

I wanted to quit at that time because of the situation that I see around me. Poor patients. I did not want to continue. Too many patient, less medical staff, less resources so I wanted to quit but having spent four years already I could not go back. There are no proper investigations, you just see patients losing their lives in the absence of medicines. **SP(F)**

### Politics

Party system and the government cannot control them. It is difficult for the students in hostel to live without associating themselves with a political party group. **RM(M)**

### Counselling:

Participants also identified lack of support from their institution in terms of counselling and career advice during studies

There is a serious crisis looming on us being a female I have so many responsibilities I do not know at this stage if I will be able to practice …… I want to have a rest, I am tired but there is no guidance now or before from seniors or options that I could have. **TT(F)**

### Learning environment:

While most of the comments were negative in nature there were a few that acknowledged the hard work that the teachers put into educating tomorrow’s doctors.

Our teachers were really great and will teach us in a good way. If we do not understand anything they will teach us again and again to get our concepts right. **NB(F)**

### Stabilization Stage

*In this phase, the participants discussed their career intentions and if they have changed since they entered the medical school. They were also asked to identify the factors that they felt would enhance retention of medical doctors in workforce*.

### Consistency of specialty choice:

At the time of entry into the medical course, fourteen participants were unsure as to which field they would pursue after graduation. Only one participant had thought about becoming a General Practitioner because there was no doctor in his area, while six had thought of Gynaecology or Paediatrics. Five participants thought of becoming surgeons, one participant had narrowed down the choices to Radiology/Psychiatry or Paediatrics while another one thought of either OBGYN or Paediatrics. These choices however, changed considerably after graduation.

Two female participants (related to each other) had earlier thought of becoming an ophthalmologist because their family runs an eye hospital. However, by the time they had graduated they started thinking of Gynaecology because it is considered a safe option and the family hospital could be extended to include a maternity section. Only three participants were consistent in their initial specialty choice while more were inclined towards Paediatrics.

All the participants except one who came for discussion wanted to continue practice.

### Influencing Factors

*A range of themes was identified by the participants that would improve the retention of the medical graduates in the workforce in Pakistan. Additionally, there were specialty specific comments that participants made. Each theme is presented separately*.

### Safety and Security

The prevailing law and order situation in Pakistan is worsening every day and was reflected in the comments.

The whole environment is deterring. …..…. From the nursing staff to patients’ family every one treats you badly. You are all the time scared of your own safety. **LB(F)**

### Social setup

Once graduated, medical graduates are more conscious of the socio-cultural perspective in which they will practice and their intentions to choose a particular field lies within that context.

…there is also a doubt among girls’ mind that when they get married will the family allow them to practice surgery because there are too many males in this field and you do not want that after spending so many years you are not allowed to practice. **AS(F)**

### Stereotyping

Beside socio-cultural norms there are gender specific and specialty specific stereotypes that could be identified from the comments which affect the decision making.

…girls cannot survive in male oriented field. The general concept is that male surgeon are more reliable than females surgeons. This is patient psychology and our society also do not prefer. **NB(F)**

### Stress

Although stress was identified as a separate theme, it was obvious from the comments that a number of other factors both at a professional and personal level, interplay to cause stress among the medical graduates.

even your family members are exhausted of your studies, and when you are the only one or one of two kids it is very difficult for parents ….Now I have my house job and then I plan for post graduation so another four years till I specialise. They will just be lonely and specially if one of them is not feeling well it is really very difficult. **AW(F)**

### Salary

By the time a student graduates, he/she is often in their mid-twenties. In the case of house officers there is a paid stipend, but if one of them is completing their house job at an institution, other than their parent institution, no stipend is paid.

During postgraduate training, a stipend is provided if a trainee is enrolled in a major training program. No stipend is paid for any other postgraduate diploma while the trainees are required to work full time and they are prohibited from taking up any employment.

…our pay is so meagre even when you have entered postgraduate training. By the time we will do post graduation and following that another 2 -3 years to get established. This means at the age of 40 years a person will be stable. There is no future except a mere title of Doctor …**RA(F)**

### Supervision

Quality of supervision was another theme identified with both positive and negative perceptions. While effective supervisors can motivate students towards a particular specialty, it was also highlighted that the graduates specifically house officers had very little contact with supervisors or senior colleagues. Postgraduate trainees who did not feel adequately supervised identified the same issue.

..in fourth year I decided to go to Ophthalmology and the reason for this is because of good teachers and the wards and the practice…. they tried to motivate us by saying that there are not many female specialists and unlike obgyn it is not saturated. **SL(F)**

### Specialisation

Participants acknowledged that specialisation is essential as a basic medical degree is just a license to practice but there are barriers that stop graduates from entering postgraduate training.

For a trainee who is enrolled in a minor diploma spread over two years training there is nothing. These trainees have to live in hostels because the training institutions are mainly in cities. This means that they have to pay hostel charges as well as for food. At the same time one is not allowed to do any part time job as part of the contract that all trainees have to sign. This is really a difficult position for boys and very unrealistic too. **SK (F)**

### Saturation

There is a realization among participants that the number of doctors produced each year is huge with more and more medical colleges starting up each year. As many of the doctors practice in the metropolitan area there is a saturation of doctors, in the area.

…there are too many medical colleges in Pakistan and even in Sindh. I can see situation of doctors not getting good jobs, there is even less respect because of saturation. Patients have easy access to doctor, if you will not see we will go to next one, you feel that these fields are over saturated **DD(M)**

### System improvement

The participants were dissatisfied with the current healthcare system in Pakistan and they identify it as being devoid of any clear policies. Similarly, the participants suggested to adapt a collaborative approach by different key organisations.

### Specialty specific factors

The number of specialties that a graduate can choose is large while the exposure to these specialties is limited. On the other hand, the participants felt that within these specialties there were specific factors that are unique to those specialties for example, lack of night duties, prognosis, working environment, community attitudes etc.

## DISCUSSION

The theory of planned behavior assumes that people consider the implications of their behavior before they decide whether to engage or not in a behavior. In the context of the medical course in Pakistan, this study has demonstrated that all three global constructs are associated with intentions to practice. However the magnitude of these constructs differ at different stages of the education. The behavioral beliefs are dominant when one enters the medical field, while control beliefs do not play any role at this stage, which is justified because these students have not really experienced what it will be like when they are practising in the field.

As the medical course progresses, normative and control beliefs overcome the behavioral beliefs. The students experience clinical rotations, observe interactions between patients and health professionals, as well as, between members of the health care team. There are both positive and negative aspects of practice experienced in this stage.

Once the graduates are in the real life situation their decisions to persue a career are mainly influenced by the control beliefs as they experience the harsh, as well as the pleasant realities of the profession and what the journey actually entails for them as professionals in the long run. The role of behavioral beliefs during this stabilisation stage is virtually non-existent.

New themes have emerged i.e. Politics and Safety, which are not reported earlier in studies elsewhere. Students who lived in hostels identified the influence of political parties through student wings predominantly. The issue of mistreatment from seniors, as well as, other members of the health professional team (paramedics) emerged along with patients and careers which is also a new theme related to safety within work environment. An earlier study has identified quality of educational program; salary structure, and poor work environment as the factors reasons for overseas migration but Politics and Safety did not emerge as themes.^9^ Similarly stress was not perceived to be an important influencing factor for those who selected surgical specialties in Pakistan,^10^ however in this study stress is an important theme.

The variation in the beliefs during the various stages supports Ajzen’s argument that the three TPB considerations can vary according to people’s situation.^11^ In another study, Arnold et al. also concluded that there was variation in the three domains within different groups^6^, and this was also supported in this study during the transition from student life to a medical graduate.^12^

While the normative beliefs are difficult to alter, policy makers and institutions need to examine how they could intervene, at various levels of training, so as to facilitate the retention of medical graduates in Pakistan.

## CONCLUSION

The analysis of data from the focus groups confirms the theoretical framework and identifies as a range of influencing factors, at different stages of education and practice. As the study was limited to a smaller number of graduates and all except one graduate wanted to continue practice, a larger sample may be required for the purpose of generalization of the findings reported in this study.
